# Investigating the microbial properties of sodium alginate/chitosan edible film containing red beetroot anthocyanin extract for smart packaging in chicken fillet as a pH indicator^☆^

**DOI:** 10.1016/j.heliyon.2023.e18879

**Published:** 2023-08-05

**Authors:** Milad Ranjbar, Mohammad Hossein Azizi Tabrizzad, Gholamhassan Asadi, Hamed Ahari

**Affiliations:** a Department of Food Science and Technology, Science and Research Branch, Islamic Azad University, Tehran, Iran; b Professor of the Department of Food Science and Technology, Tarbiat Modares University, Tehran, Iran; c Assistant Professor of the Department of Food Science and Technology, Science and Research Branch, Islamic Azad University, Tehran, Iran; d Professor of the Department of Food Science and Technology, Science and Research Branch, Islamic Azad University, Tehran, Iran

**Keywords:** Smart packaging, Anthocyanin, pH sensor, Microbial load, Chicken fillet

## Abstract

The current trend in the production of smart films involves the use of pH-responsive color indicators derived from natural sources. In line with this trend, the aim of this research is to produce edible films from sodium alginate (A) and chitosan (Ch) incorporating red beet anthocyanin (Ac) extract, and to assess the properties of these films and their use as coatings for chicken fillets. The study employed a factorial design to evaluate the effects of treatments C (control), A25%-ch75% (films consisting of 25% sodium alginate and 75% chitosan), and A25%-ch75%-Ac (films consisting of 25% sodium alginate, 75% chitosan, and red beet anthocyanin). The findings indicate that the inclusion of red beet anthocyanin extract did not result in any discernible differences in the FTIR spectra of the film samples. Analysis of the XRD results revealed that the addition of the extract led to a reduction in the crystal structure of the film. Moreover, SEM results demonstrated that the extract caused alterations in the polymer chains and an increase in the porosity of the film matrix. With regard to the chicken fillet samples coated with the film, over time, there was an increase in microbial analysis (total microorganism count and Staphylococcus aureus coagulase-positive) and chemical properties (pH, peroxide, thiobarbituric acid, and nitrogen compounds) for all samples. However, this trend was significantly lower in the samples coated with the Ac extract (P < 0.05). Texture analysis results revealed that the hardness parameter of all samples decreased over the storage period, while the samples containing the Ac extract demonstrated a significant increase in this parameter (P < 0.05). Additionally, the color changes of the pH sensor corresponded to the anthocyanin structure. Based on the results, the smart film composed of sodium alginate/chitosan incorporating red beet anthocyanin extract has the potential to enhance the quality, prolong the shelf life, and decrease the microbial load of chicken fillet when used as a coating. Furthermore, red beet anthocyanin can serve as a suitable indicator for spoilage changes in packaged food products.

## 1 Introduction

The packaging of food products is an essential process in the production of food items, with a crucial role in guaranteeing their quality and safety [1]. In response to the growing awareness of consumers and the demand for fresh and safe food products, novel technologies have emerged [[2], [3], [4]]. Smart packaging, featuring natural color indicators, has garnered significant attention in this context, owing to its ability to monitor the food condition, provide information on its chemical, biochemical, physical, and microbiological quality, as well as its accessibility, wide availability in nature, cost-effectiveness, and non-toxicity compared to artificial colorants [5,6]. Anthocyanins belong to the phenolic family of active bioactive compounds and are the most essential plant pigments. The six prevalent types of anthocyanins are pelargonidin, cyanidin, malvidin, peonidin, delphinidin, and petunidin [7,8]. These compounds possess a C6C3C6 carbon skeleton with a flavilium ion or 2-phenyl-benzopyrylium structure [9]. The favorable characteristics of natural anthocyanins include attractive coloration, high water solubility, high biological activity, low toxicity, easy and rapid accessibility, and affordability. Furthermore, they are utilized as food additives to improve human health, owing to their anti-inflammatory, antioxidant, anti-obesity, anti-aging, anticancer, antidiabetic, and antimicrobial, neuroprotective, and immune system-enhancing properties [10,11]. According to the studies done by different researchers on the effects of anthocyanin compounds, showed that anthocyanins are known to exert a significant impact on the functional and physical properties of the substance under investigation. In this study, the source of anthocyanins was identified as red beetroot extract, derived from Beta vulgaris, a plant belonging to the spinach family. The plant comprises two groups of pigments, namely betacyanins and betaxanthins, which impart reddish-purple and yellow hues, respectively. Betalains are water-soluble glycosides that resemble anthocyanins and flavonoids in appearance. Betanin, the primary pigment in beets, is the most important betacyanin [12]. Red beetroot and the extract obtained from it have several bioactive compounds with antioxidant, coloring and stabilizing properties [13]. Also the red beetroot and the extract of red beetroot have been found to offer preventive and therapeutic effects against various diseases, such as hypertension, heart disease, different types of cancer, blood lipid disorders, diabetes, liver disorders, infectious diseases, weakness, anemia, gastrointestinal diseases, stomach ulcers, and constipation, among others. Additionally, red beets exhibit a high capacity for scavenging free radicals and, in low concentrations, can prevent lipid peroxidation and reduce liver enzyme activity [14,15].

Chitosan, a cationic natural carbohydrate polymer composed of 2-amino-deoxy-β-D-glucan linked by (1,4) and obtained by deacetylation of chitin, exhibits cationic behavior in acidic solutions due to the protonation of its amino groups [16,17]. The numerous desirable properties of chitosan, such as availability, naturalness, and antifungal and antibacterial properties, have made it a popular choice in various fields, including cosmetics, healthcare, food industry, and pharmaceuticals. One of the potential applications of chitosan is as an edible coating due to its excellent film-forming properties, durability, flexibility, ability to modify the internal atmosphere, and reduction of moisture loss [18,19].

Alginate is a water-soluble anionic linear polysaccharide that is extracted from the cell walls of brown seaweed (Laminaria digitata/Ascophyllum nodosum) and synthesized by microorganisms. Some of the most notable properties of alginate include its ability to form gels, low solubility polymers, improved mechanical properties, barrier properties, and consistency. Sodium alginate, a non-toxic, biodegradable, biocompatible, and cost-effective hydrocolloid, has numerous applications in different fields such as packaging to reduce dehydration of meat, gelling agent, colloidal stabilizer in the beverage industry, textiles, pharmaceuticals, and polymer matrices for encapsulating drugs, proteins, cells, and DNA [20,21].Over the past few years, a multitude of studies have been conducted to explore the use of anthocyanins as indicators in food films. These films are designed to monitor the quality of packaged products and may include smart films based on various materials, such as chitosan/chitin nano-crystals containing anthocyanins from red cabbage [22], starch-based films, tara gum and polyvinyl alcohol containing anthocyanins extracted from the skin of black mulberry for preserving tilapia fillets [23], starch-based smart films containing agar/potato and anthocyanin extract from purple sweet potato in pork packaging [24], pectin-based watermelon rind and purple cabbage anthocyanin films for assessing the freshness of lamb meat [25], nano-fibrous films based on chitosan/polyethylene oxide containing natural curcumin [26], smart film based on cassava starch containing red cabbage extract among others [27]. Despite the considerable research on this topic, there has been no investigation into the use of red beet anthocyanin extract as a pH indicator in food films based on sodium alginate/chitosan. Thus, this study aims to fill this gap by examining the effect of red beet anthocyanin extract on the structural properties of smart films and assessing the chemical and microbiological properties of packaged chicken fillets during storage. The hypotheses of the present study can be expressed as follows: the use of red beetroot anthocyanin extract leads to the improvement of the structural properties of edible films, and also the addition of Ac extract leads to improved quality, increased shelf life and reduced microbial load of packaged chicken fillets.

## 2 Materials and methods

### 2.1 Materials

The red beetroot and chicken fillet were procured from the local market in Sanandaj, Iran. The chemicals used in the study included glycerol with a purity of 99.5%, sodium alginate with a molecular weight of 1.93 × 105 g/mol, chitosan with an average molecular weight, a purity of 99%, and a degree of deacetylation of 70%, and other chemicals, all of which were procured from the Merck brand based in Germany.

### 2.2 Preparation of red beetroot extract

The current study utilized a modified method of red beetroot extract preparation, based on the technique reported by Ghasempour et al. (2020) [28]. Initially, fresh red beetroot was cleaned, peeled, and cut into small pieces. The resulting beetroot pieces were uniformly and superficially dried in the shade, followed by grinding using a domestic grinder (Moulinex model AR110, France). Subsequently, 250 g of the obtained powder was mixed with a solvent composed of 250 mL of ethanol/water (30% v/v) containing 1.5% citric acid. The mixture underwent sonication for 15 min at 60 °C, followed by 45 min at 25 °C using a Hielscher UP400ST sonicator (Germany). After blanching, the mixture was centrifuged at 4000 rpm for 15 min. The upper phase containing ethanol was collected and removed using a Heidolph Laborata 4003 rotary evaporator (Germany). Ultimately, the aqueous solution obtained from the process was utilized as red beetroot extract for packaging films.

### 2.3 Preparation of smart films

The preparation of control smart films involved a stepwise process. Initially, 0.75 g of sodium alginate was dissolved in 100 mL of distilled water for 40 min at 70 °C on a magnetic stirrer to improve dissolution. Then, 2.25 g of chitosan was mixed with 100 mL of distilled water containing 0.7% acetic acid on a magnetic stirrer at 40 °C. The resulting chitosan solution was filtered with a Whatman filter paper to remove insoluble impurities. Subsequently, the sodium alginate solution was slowly added to the chitosan solution, and the mixture was stirred for 15 min on a magnetic stirrer. Glycerol was added to the solution at a level of 6% of the dry weight, followed by stirring for an additional 15 min. The resulting solution was poured onto a plate, air-dried for 10 min, and a specific amount of the solution was cast onto another plate and dried at 40 °C. The film was then peeled off from the plate. Then, to investigate the effect of red beetroot extract as an anthocyanin compound in the packaging film; adding approximately 25 mL of the extract to the film and homogenizing for 2 min at a speed of 12,000 rpm using a homogenizer (Heidolph D9I12, Germany) [21,29].

### 2.4 Application of the films as smart labels

To make a smart label, 2 layers of control film with dimensions of 6 × 5 cm were selected, then 5 mL of substance sensitive to pH change (red beetroot extract) was added to it, and then it was connected by thermal glue and placed inside the package.

#### 2.4.1 Tests

##### 2.4.1.1 Measurement of anthocyanin concentration using pH differential method

To determine the concentration of anthocyanin in the red beetroot extract, the pH differential method was used. Firstly, 5 mL of the extract was mixed with 20 mL of pH 1.0 buffer (0.25 M potassium chloride) and left to incubate for 20 min at room temperature. The mixture was then centrifuged at 4000 rpm for 15 min, and the resulting supernatant was measured for absorbance at 520 and 700 nm wavelengths. The same process was repeated using pH 4.5 buffer (0.4 M sodium acetate). The anthocyanin concentration was finally calculated in milligrams per gram, based on the provided formula [30], where V represents the volume of extract used:

Anthocyanin concentration = [(A520nm - A700nm) pH1.0 - (A520nm - A700nm) pH4.5] × V.

### 2.5 Measurement of anthocyanin concentration using chromatography method

The concentration of anthocyanin in the red beetroot extract was determined using an HPLC system (Boeblingen, Germany) that included a quaternary pump, a diode array detector operating at 530 nm, ChemStation software, an autosampler, and a C18-packed column (250 mm × 4.6 mm, Japan). The mobile phase consisted of 5% formic acid/water as solvent A and 5% formic acid/acetonitrile as solvent B. The column temperature was maintained at 30 °C, and 21 μL was injected for each experiment, with a flow rate of 0.5 mL/min. The anthocyanin concentration was determined in milligrams per gram by comparing the HPLC peak areas with standard calibration curves [31].

#### 2.5.1 FTIR test

The samples were prepared as thin discs using the potassium bromide method with a thickness of less than 1 mm. The Fourier Transform Infrared (FTIR) spectrophotometer (PerkinElmer, USA Spectrum Two) was utilized to analyze the samples in the range of 450–4000 cm-1 with a resolution of cm-11. This test was performed to investigate new bonds, compound interactions, and molecular properties [32].

#### 2.5.2 XRD test

An X-ray device (Bruker AXS, Karlsruhe, Germany) was used to examine the crystalline structure of the films. The device was set at 40 kV and 30 milliamps, and the samples were scanned from 2 to 70° at a speed of 1° with intervals of 5/0°. Additionally, the wavelength of nm1539/0 was applied during the test. This analysis was conducted to provide insights into the films' structural properties [32].

#### 2.5.3 SEM test

The scanning electron microscope (SEM) (Philips, Netherlands) was employed to investigate the microstructure and morphology of the produced films. To prepare the samples for imaging of the cross-sectional surface, they were fractured in liquid nitrogen, coated with particles for 40 s, and attached to a metal base with double-sided carbon tape. The imaging was performed at a magnification of 500 times. This test was conducted to gain insights into the microstructural properties of the films [33].

#### 2.5.4 pH

The pH measurement of chicken fillet samples was carried out at 25 °C using a digital pH meter (Model 827, Metrohm, Switzerland) following calibration with buffer solutions having pH values of 7 and 4. Homogenization of 10 g of chicken fillet samples with 100 mL of distilled water for 60 s was performed using a homogenizer. The pH value of the samples was then determined [34].

#### 2.5.5 Peroxide

The peroxide value of packaged chicken fillet samples was determined using the standard method (AOAC, 2005). Initially, 0.3 g of chicken fillet samples were mixed with 8.9 mL of chloroform-methanol (1:1) by vortexing for 5 s. A volume of 0.05 mL of 10 mM ammonium thiocyanate solution was then added to the mixture and vortexed for another 5 s. To this mixture, 0.5 mL of iron (II) solution was added and vortexed for 5 s. The resulting mixture was incubated at room temperature for 5 min, and the absorption was recorded at 500 nm wavelength.

### 2.6 Nitrogenous volatile compounds

The nitrogenous volatile compounds of chicken fillet samples were quantified using the Kjeldahl method. To this end, 10 g of chicken fillet samples, 2 g of magnesium oxide, 500 mL of distilled water, and several pieces of boiling stones were added to a Kjeldahl flask. The mixture was then subjected to distillation using the Kjeldahl apparatus. The resulting distillate was added to a solution containing 2% boric acid and a few drops of methyl red as an indicator. Finally, the yellow solution obtained was titrated with 1.0 N sulfuric acid until the appearance of pink color. The content of nitrogenous volatile compounds was expressed as milligrams of nitrogen per 100 g of chicken fillet sample [35].

Sulfuric acid consumption volume x 14 = Volatile Nitrogen Compounds (TVN).

#### 2.6.1 Thiobarbituric acid

In order to determine the thiobarbituric acid index, a mixture of 10 g of chicken fillet sample, 35 mL of 5% normal trichloroacetic acid (TCA), and 1 mL of 1% butylated hydroxytoluene (BHT) was homogenized at 100 rpm for 2 min and filtered through a Whatman filter paper. The resulting solution was then diluted with TCA to a final volume of 50 mL. Subsequently, 5 mL of the diluted solution were mixed with 2.0 M thiobarbituric acid (TBA) and subjected to heat in a boiling water bath at 100° for 1 h. The absorption at a wavelength of 532 nm was measured using a spectrophotometer (Helios, England), and the TBA value was calculated using the equation TBA = (50 × (Abs – Ab))/200 [36].

##### 2.6.1.1 Microbial analysis

In order to determine the microbial load of packaged chicken fillet samples, a homogenate of 10 g of chicken fillet in 90 mL of 1.0% peptone water was prepared using a stomacher for 60 s. Sequential dilutions were then prepared using 1.0% peptone water. For the enumeration of total microorganisms, 1 mL of each dilution was added to Nutrient Agar and incubated at 37 °C for 24 h. The bacterial growth was reported as log10 CFU/g [8,37]. For the enumeration of Staphylococcus aureus, 1.0 mL of the dilutions was added to Mannitol Salt Agar and incubated for 24–48 h at 37 °C, and colonies were counted using a colony counter [38,39]. To detect the presence of Salmonella, 10 g of chicken fillet with 90 mL of 1% peptone water were placed in a sealed container and incubated at 37 °C for 18–20 h. Subsequently, 1 mL of the primary culture was added to 9 mL of selenite cystine broth and incubated at 37 °C for 24–48 h. After the incubation period, a linear streak was made on Shigella Salmonella Agar and incubated at 37 °C for 24 h [40].

#### 2.6.2 Texture hardness

To determine the texture hardness of chicken fillet samples, a texture analyzer (TA-XT Plus, Stable Micro System Ltd, Surrey, UK) was utilized. Samples measuring 6 × 4 × 4 cm were prepared from the central portion of the fillets for the test. The texture test was performed using a dual compression method, with a 5-s time interval between two cycles, and a 75-mm diameter probe. The device was configured to a test speed of 2 mm per second, and pre-test and post-test speeds of 3 mm per second, with a 50% compression rate applied [41].

### 2.7 Color indicator changes under acidic and basic conditions

To investigate color indicator changes under acidic and basic conditions, phosphate buffer solutions with different pH values were prepared using a mixture of phosphoric acid and sodium hydroxide. The color indicators, with dimensions of 3 × 3 cm, were immersed in the buffer solutions for 5 min at room temperature [8,42].

### 2.8 Statistical analysis

The data obtained from the experiment were statistically analyzed using a completely randomized factorial design. The SPSS version 26 software was used for this purpose. All tests for each group were conducted in triplicate. Two-way ANOVA was employed at a 95% confidence level for statistical comparison. For drawing graphs, Excel software was used, and for mean comparison, Duncan's test was utilized. All results were presented in the form of mean ± standard deviation. Also before the analysis, the Kolmogorov Smirnov test was used in the SPSS software and the results indicate the normality of the data.

## 3 Results and discussion

### 3.1 Anthocyanin concentration by pH differential and chromatography methods in red beet extract

The results of anthocyanin concentration in red beet extract are presented in Table 1. Based on the findings of this study, the measured anthocyanin concentration using the HPLC method is higher than that obtained by the pH differential method due to its higher accuracy.

**Table 1:** Comparison and analysis of anthocyanin concentration by pH differential and chromatography methods in red beet extract.

Anthocyanin Content Measured by Chromatography	Amount of Anthocyanin in Relation to pH Differences
232.61 ± 0.87	226.5 ± 0.898

### 3.2 SEM results of beetroot anthocyanin-containing film

**Fig. 1:**
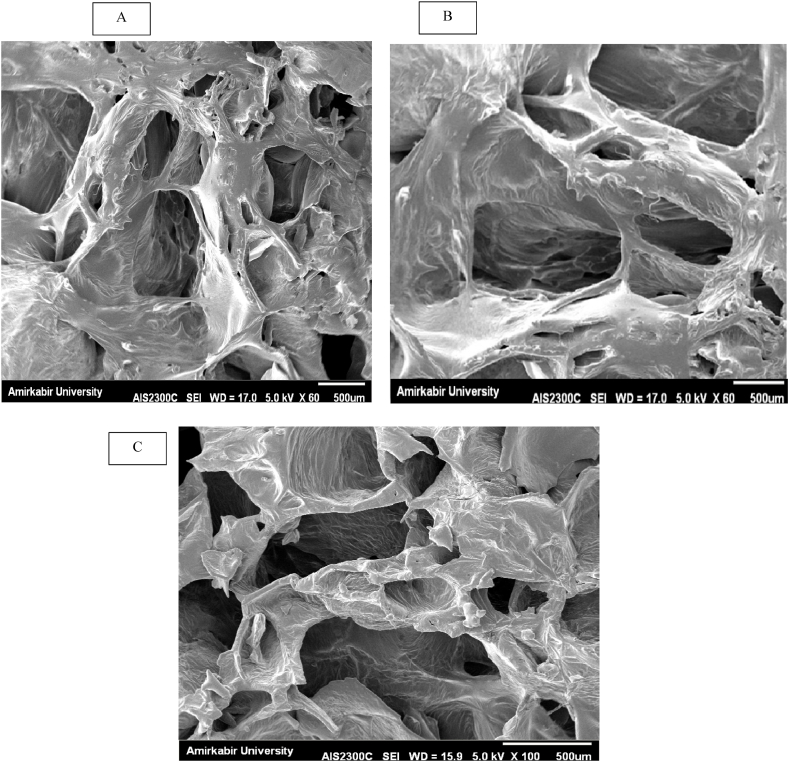
Scanning Electron Microscopy Image of A: Control sample, B: 25% Sodium Alginate and 75% Chitosan, C25% Sodium Alginate, 75% Chitosan, and red beet anthocyanin extract Film.

Fig. 1 displays electron microscopy images that illustrate the cross-sectional surface of contain control film (A), film without beetroot anthocyanin extract (B) and film containing beetroot anthocyanin extract (C). Scanning electron microscopy (SEM) observations revealed that the sodium alginate/chitosan film exhibited a uniform network structure with a continuous and homogeneous phase in the matrix. However, upon the addition of red beetroot anthocyanin extract, alterations in the polymer chains occurred, leading to an increase in the porosity of the film matrix [43]. These findings are in agreement with those reported by Bitencourt et al. (2014), who observed similar modifications in the microstructure of a gelatin-based film upon incorporating curcumin extract [44].

### 3.3 FTIR results of beet anthocyanin-containing film

**Fig. 2:**
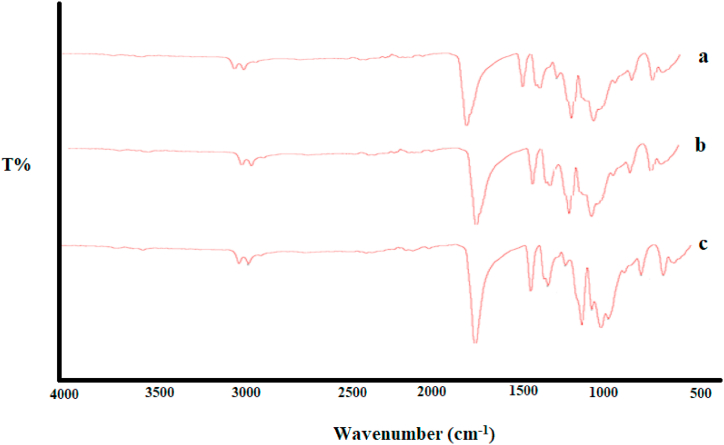
FTIR Spectrum of a: Control sample, b: 25% Sodium Alginate and 75% Chitosan, c 25% Sodium Alginate, 75% Chitosan, and red beet anthocyanin extract Film.

In Fig. 2, the film sample contain control film (A), film without beetroot anthocyanin extract (B) and film containing beetroot anthocyanin extract (C) are analyzed using Fourier-transform infrared (FTIR) spectroscopy. The FTIR spectrum of the samples showed absorption patterns in the range of 600 cm-1 to 4000 cm-1, as shown in Fig. 2. The results indicated no significant differences in the FTIR spectrum of the film samples upon the addition of beet anthocyanin extract, suggesting electrostatic interactions between the beet anthocyanin extract and the sodium alginate/chitosan matrix. The peak at 2944-2994 cm-1 was attributed to the stretching vibrations of the C–H bonds in the chitosan polymer chain [45]., while the peak at 1745 cm-1 was associated with the stretching vibrations of the C=O bonds in methyl carboxyl groups [46]. The peak at around 1149 cm-1 was related to the stretching and vibrational modes of the carboxyl groups in the sodium alginate and the C=C aromatic ring stretching vibrations. Peaks in the range of 1200 cm-1 to 1370 cm-1 indicated C─O stretching vibrations in the polysaccharide mixture [47,48]. Additionally, the peak in the range of 650 cm-1 to 950 cm-1 was attributed to the presence of C=C and C─H bonds [49]. The peak in the range of 1000 cm-1 to 1180 cm-1 was related to the vibrational modes and connections of the C–O–C bonds. These findings provide insights into the chemical composition and interactions of the beet anthocyanin-containing film [50].

### 3.4 XRD results of beet anthocyanin-containing film

**Fig. 3:**
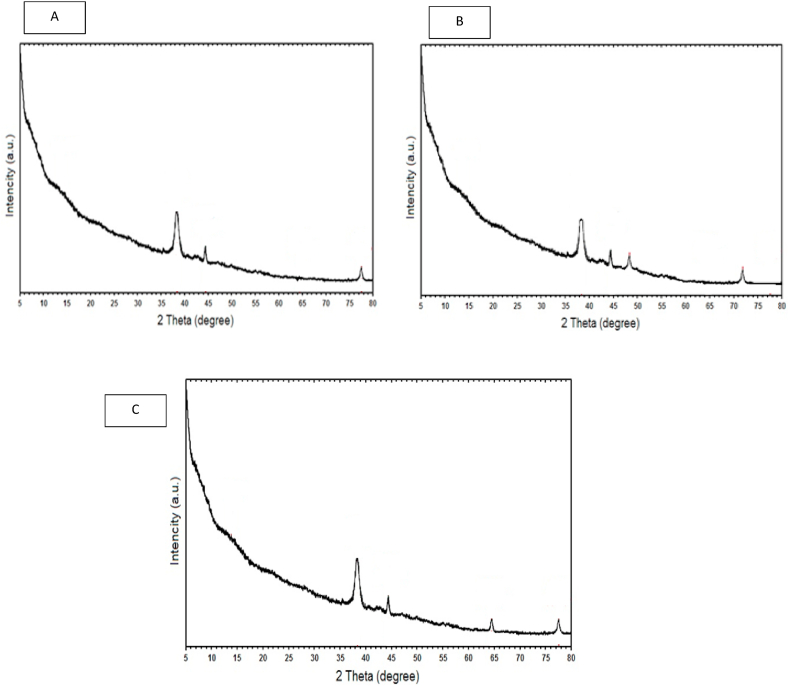
X-ray Diffraction Pattern of A: Control sample, B: 25% Sodium Alginate and 75% Chitosan, C25% Sodium Alginate, 75% Chitosan, and red beet anthocyanin extract Film.

The crystal structure of control film (A), film without beetroot anthocyanin extract (B) and film containing beetroot anthocyanin extract (C) was investigated using X-ray diffraction (XRD), as shown in Fig. 3. The XRD spectrum of the samples showed characteristic peaks at approximately 11.14°, 26.38°, 47.44°, 71.64°, and 74.77°, as shown in Fig. 3. Diffraction peaks at 26/38, 47/44 are the main characteristics of chitosan and sodium alginate. These results show that the addition of red beetroot anthocyanin extract leads to an increase in the amorphous form in the alginate/chitosan edible film. From these results, it can be concluded that by pairing anthocyanin, the intra- and intermolecular hydrogen bonds are broken and the degree of crystallinity decreases. In other word the results indicated that the addition of beetroot anthocyanin extract to the sodium alginate/chitosan film did not result in significant shifts in the peak positions. However, the crystal structure of the film was somewhat affected, as evidenced by the reduction in the peak intensity. These findings provide insights into the chemical and physical interactions between the beetroot anthocyanin extract and the sodium alginate/chitosan matrix. Similar results were reported by Shabhangu et al. (2022) for the addition of betanin dye to a protein isolate/water-soluble pectin-based film [51].

#### 3.4.1 pH analysis

**Fig. 4:**
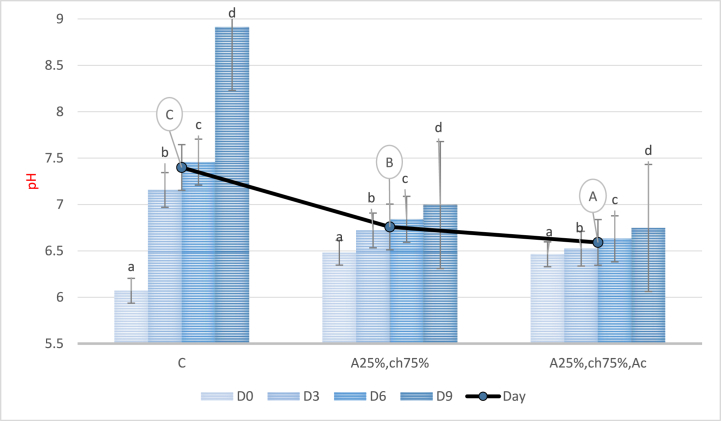
pH levels in chicken fillet coated with a sodium alginate/chitosan-based film containing red beet anthocyanin extract during storage period C: Control sample, A25%-Ch75%: Film containing 25% Sodium Alginate and 75% Chitosan, A25%-Ch75%-Ac: Film containing 25% Sodium Alginate, 75% Chitosan, and red beet anthocyanin extract. Note: Lowercase letters indicate significant differences between a treatment on different days, while uppercase letters indicate significant differences between treatments (P < 0.05).

Fig. 4 illustrates the changes in pH over a storage period of nine days. The analysis of variance revealed that the pH of all treatments increased significantly during the storage period (P < 0.05). However, in the samples coated with a sodium alginate/chitosan film containing red beet anthocyanin extract, the pH decreased significantly compared to the control and the samples without anthocyanin extract (P < 0.05). The most significant decrease in pH was observed in the samples coated with the sodium alginate/chitosan film containing red beet anthocyanin extract, whereas the control sample showed the highest pH. The increase in pH during the storage period could be due to the denaturation of protein, leading to the release of amino compounds such as ammonia, amines, dimethylamine, and trimethylamine during the breakdown of amino acids and proteins [52]. Conversely, the presence of antimicrobial and phenolic compounds in the red beet anthocyanin extract influenced the growth of spoilage bacteria, resulting in a decrease in pH [53]. Comparable findings were reported by Kanatt (2020) and Contini et al. (2022) with the addition of Amaranthus leaf extract in polyvinyl alcohol/gelatin film for the preservation of chicken/fish meat [54] and with the addition of lemon grass essential oil in chitosan film for the preservation of chicken meat [55].

#### 3.4.2 Investigation of peroxide

**Fig. 5:**
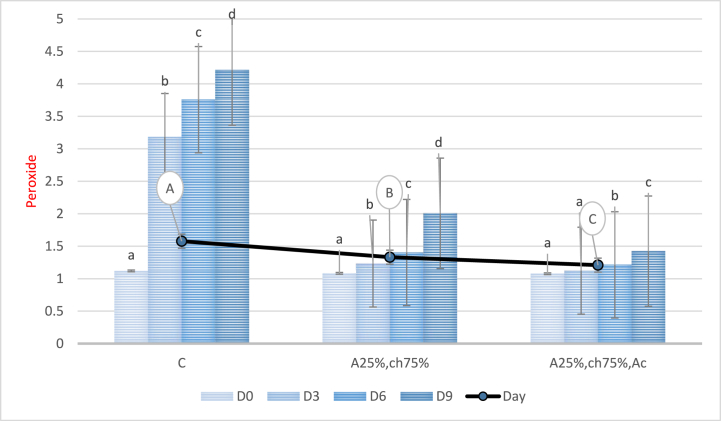
Peroxide levels in chicken fillet covered with a sodium alginate/chitosan film containing red beet anthocyanin extract during storage. C: Control sample, A25%-Ch75%: Film containing 25% Sodium Alginate and 75% Chitosan, A25%-Ch75%-Ac: Film containing 25% Sodium Alginate, 75% Chitosan, and red beet anthocyanin extract. Note: Lowercase letters indicate significant differences between a treatment on different days, while different uppercase letters indicate significant differences between treatments (P > 0.05).

The changes in peroxide levels during a 9-day storage period were examined, as presented in Fig. 5. The results of the analysis of variance indicated that the peroxide level significantly increased (P < 0.05) in all treatments during the storage period. However, the samples covered with a sodium alginate/chitosan film containing red beet anthocyanin extract showed a significant reduction in peroxide levels compared to the control and samples without anthocyanin extract (P < 0.05). The highest reduction in peroxide levels was observed in samples covered with a sodium alginate/chitosan film containing red beet anthocyanin extract, while the highest level was observed in the control sample. The observed decrease in peroxide levels can be attributed to the presence of phenolic compounds in red beet anthocyanin extract, which act as free radical scavengers and electron donors, thereby trapping free radicals, particularly peroxide radicals. This ultimately terminates the cycle of oxidative degradation reactions and reduces the rate of peroxide increase during storage [56,57]. These findings are consistent with the results of other studies that added natural extracts to packaging films to preserve food products. Bashir and colleagues (2022) added pomegranate peel powder to a sodium alginate-based film to preserve chicken nuggets [58], while Barkhordari & Bazargani-Gilani (2021) added apple peel extract and ginger essence to a zein-based film to preserve chicken leg meat [59].

### 3.5 Investigation of volatile nitrogen compounds

**Fig. 6:**
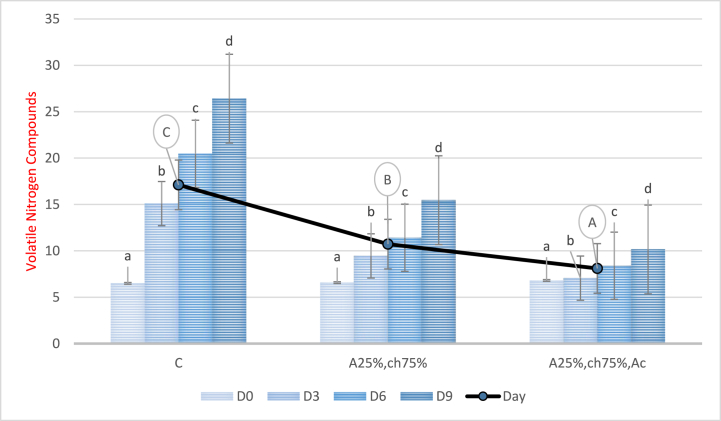
Nitrogen compounds levels released from chicken fillet covered with a film based on sodium alginate/chitosan containing red beet anthocyanins during the storage time C: Control sample, A25%-Ch75%: Film containing 25% Sodium Alginate and 75% Chitosan, A25%-Ch75%-Ac: Film containing 25% Sodium Alginate, 75% Chitosan, and red beet anthocyanin extract. Note: Lowercase letters indicate significant differences between a treatment on different days, while different uppercase letters indicate significant differences between treatments (P < 0.05).

In Fig. 6, the changes in the index of volatile nitrogen compounds during a 9-day storage period are presented. The results of the analysis of variance revealed a significant increase (P < 0.05) in the volatile nitrogen compounds index of the samples during the storage period. However, the samples covered with a sodium alginate/chitosan film containing red beet anthocyanin extract exhibited a significant decrease in this parameter compared to the control and samples without anthocyanin extract (P < 0.05). The greatest reduction in volatile nitrogen compounds was observed in samples covered with a sodium alginate/chitosan film containing red beet anthocyanin extract, while the highest level was observed in the control sample. The observed increase in volatile nitrogen compounds during storage can be attributed to the accumulation of ammonia, dimethylamine, and trimethylamine resulting from the breakdown of nitrogenous compounds [60]. On the other hand, the decrease in volatile nitrogen compounds due to the use of a film containing red beet anthocyanin extract is mainly attributed to the antimicrobial property of the factor used, which leads to a reduction in bacteria responsible for the oxidative deamination of non-protein nitrogenous compounds. The findings of this study are consistent with the results of other studies that added natural extracts to packaging films to preserve food products [61]. Boonsiriwit and colleagues (2022) used a cellulose-based biocomposite film containing roselle anthocyanin to preserve chicken fillet [62], while Esmaeli and colleagues (2019) added a combination of Pimpinella affinis extract and essence to a zein-based coating on packaged rainbow trout fillet during storage in a vacuum [63].

### 3.6 Examination of thiobarbituric acid index (TBA)

**Fig. 7:**
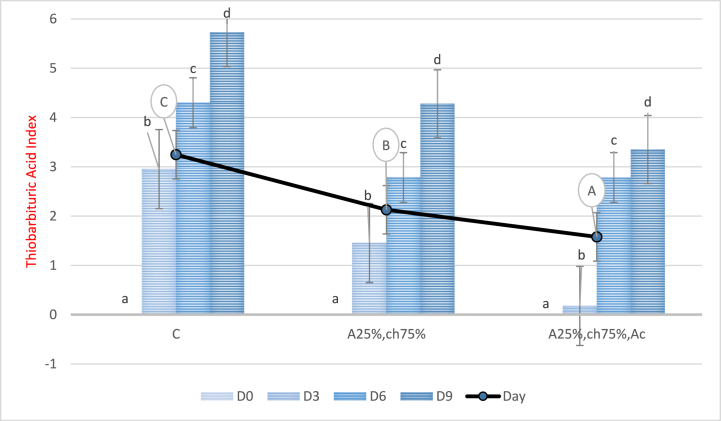
Level of TBA index in chicken breast fillets covered with a film based on sodium alginate/chitosan containing red beet anthocyanin extract during the storage time. C: Control sample, A25%-Ch75%: Film containing 25% Sodium Alginate and 75% Chitosan, A25%-Ch75%-Ac: Film containing 25% Sodium Alginate, 75% Chitosan, and red beet anthocyanin extract. Note: Lowercase letters indicate a significant difference between treatments on different days, while uppercase letters indicate a significant difference between treatments (P < 0.05).

Fig. 7 displays the TBA index results obtained during the storage period of chicken breast meat. The statistical analysis and Fig. 7 demonstrate a significant increase in the TBA index of the samples during storage (P < 0.05). However, samples coated with a film based on sodium alginate/chitosan containing red beet anthocyanin extract showed a significant reduction in the TBA index compared to both the control and samples without anthocyanin extract (P < 0.05). The lowest TBA index was observed in the samples coated with the aforementioned film, while the control sample exhibited the highest nitrogen compounds level. The elevation of TBA index during storage is attributed to lipid oxidation, the generation of volatile metabolites, and tissue hydrogenation [64]. Furthermore, red beet anthocyanin extract's antioxidant properties and phenolic compounds such as carvacrol, thymol, and methyl ether derivatives, as well as p-cymene, y-terpinene, and anthocyanidins, inhibit the initiation of free radical chain reactions, resulting in a decrease in the TBA index [51]. These findings are in line with the studies conducted by Kanatt (2020) [54], incorporating Amaranthus flower leaf extract for preserving chicken and fish meat, and Bahrami Feridoni & Khademi Shurmasti (2020) [56], using Hibiscus sabdariffa L. extract for chicken nugget preservation.

### 3.7 Total microbial count

**Fig. 8:**
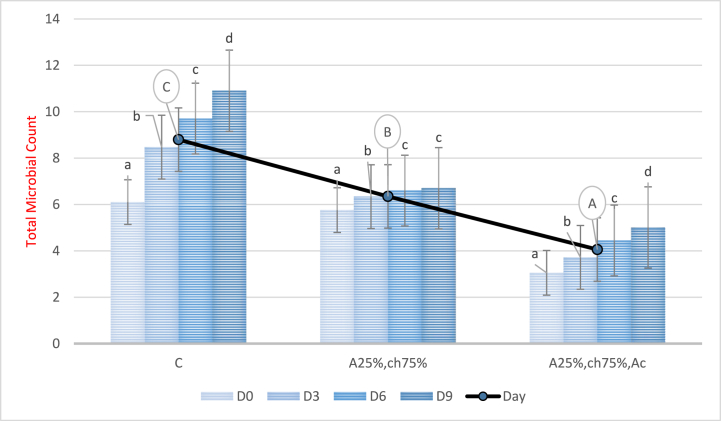
Total count of microorganisms in chicken fillet covered with a film based on sodium alginate/chitosan containing red beet anthocyanin extract during the storage period. C: Control sample, A25%-Ch75%: Film containing 25% Sodium Alginate and 75% Chitosan, A25%-Ch75%-Ac: Film containing 25% Sodium Alginate, 75% Chitosan, and red beet anthocyanin extract. Note: Lowercase letters indicate a significant difference between a treatment on different days, and different uppercase letters indicate a significant difference between treatments (P < 0.05).

Fig. 8 illustrates the total microbial count during a 9-day storage period. The results of the analysis of variance indicate a significant increase in total microbial count in all treatments over time (P < 0.05). However, the total microbial count decreased significantly in samples covered with a film based on sodium alginate/chitosan containing red beet anthocyanin extract compared to the control and samples without anthocyanin extract (P < 0.05). The lowest total microbial count was observed in samples covered with the aforementioned film, while the highest count was observed in the control sample. The observed reduction in microbial load in coatings containing red beet anthocyanin extract is attributed to its antimicrobial property. These findings are consistent with the results of Turan & Şimşek's (2021) study, which demonstrated that the total microbial count of beef stored in aerobic and vacuum packaging decreased during the storage period due to the antimicrobial property of blackberry anthocyanin extract [65].

### 3.8 count of staphylococcus aureus coagulase-positive

**Fig. 9:**
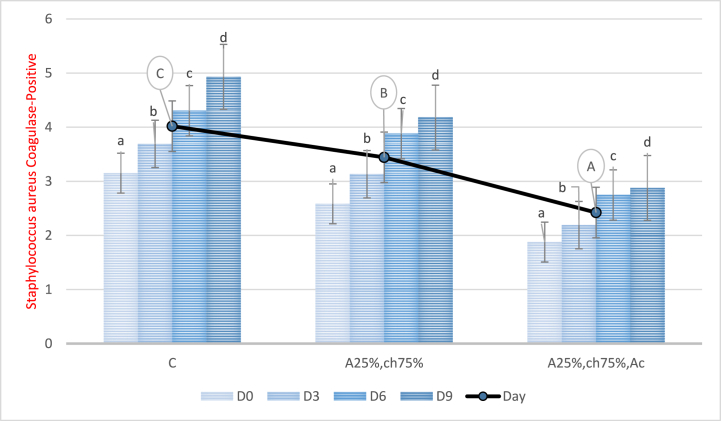
Count of Staphylococcus aureus coagulase-positive in chicken fillet covered with a film based on sodium alginate/chitosan containing red beet anthocyanin extract during the storage period. C: Control sample, A25%-Ch75%: Film containing 25% Sodium Alginate and 75% Chitosan, A25%-Ch75%-Ac: Film containing 25% Sodium Alginate, 75% Chitosan, and red beet anthocyanin extract. Note: Lowercase letters indicate a significant difference between a treatment on different days, and different uppercase letters indicate a significant difference between treatments (P < 0.05).

Fig. 9 displays the effect of a film made from sodium alginate/chitosan and red beet anthocyanin extract on the count of coagulase-positive Staphylococcus aureus in chicken fillet during storage. The results revealed a significant upward trend (P < 0.05) in the bacterial population in all treatments during the storage period. However, the count of Staphylococcus aureus coagulase-positive in samples covered with the film containing red beet anthocyanin extract significantly decreased compared to the control and non-extract samples (P < 0.05). Notably, the lowest count of Staphylococcus aureus coagulase-positive was observed in the extract-containing film samples, while the highest count was related to the control sample. These findings indicate the antimicrobial property of red beet anthocyanin extract. The current study's results are consistent with those reported by Tometri et al. (2020), who used Laurus nobilis leaf extract in minced meat storage [66], and Mahdavi et al. (2018), who used Pimpinella anisum L essential oil in chitosan-based film packaging for chicken burgers [67].

### 3.9 Salmonella count

Table 2 displays the mean Salmonella count in chicken fillet covered with different treatments during the storage period. The statistical analysis showed no significant difference in the Salmonella count among the treatments (P > 0.05). In the other word no differences were found in all values below the detection limit (<100). Also these results suggest that the applied treatments did not have a significant impact on the Salmonella count in the chicken fillet during the storage period.

**Table 2:** Logarithmic values of Salmonella count in chicken fillet covered with different treatments during the storage period.

Treatment	Day 0	Day 3	Day 6	Day 9
**C**	< 100	< 100	< 100	< 100
**A25%-ch75%**	< 100	< 100	< 100	< 100
**A25%-ch75%-Ac**	< 100	< 100	< 100	< 100

C: Control sample, A25%-Ch75%: Film containing 25% Sodium Alginate and 75% Chitosan, A25%-Ch75%-Ac: Film containing 25% Sodium Alginate, 75% Chitosan, and red beet anthocyanin extract.

### 3.10 meat texture hardness

**Fig. 10:**
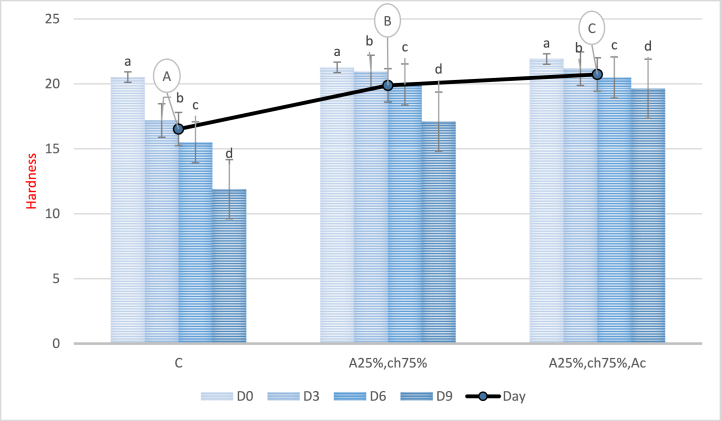
Hardness of chicken fillet covered with Sodium Alginate/Chitosan film containing red beet anthocyanin extract during the storage period. C: Control sample, A25%-Ch75%: Film containing 25% Sodium Alginate and 75% Chitosan, A25%-Ch75%-Ac: Film containing 25% Sodium Alginate, 75% Chitosan, and red beet anthocyanin extract. Note: Lowercase letters indicate significant differences between a treatment on different days, while different uppercase letters indicate significant differences between treatments (P > 0.05).

Fig. 10 displays the evolution of chicken fillet hardness during storage. The study found a statistically significant decrease in fillet hardness for all treatments over time (P < 0.05). However, the fillet hardness was significantly increased in the samples covered with the sodium alginate/chitosan film containing red beet anthocyanin extract compared to the control and samples without anthocyanin extract (P < 0.05). The highest hardness was observed in the samples covered with the film containing sodium alginate/chitosan and red beet anthocyanin extract, while the lowest hardness was related to the control sample. Hardness is a crucial quality attribute for food products, and a soft texture is not desirable for certain foods, such as meat, from the consumer's perspective. The use of the sodium alginate/chitosan film containing red beet anthocyanin extract for packaging resulted in increased hardness of the samples compared to other treatments, which may be attributed to lipid/protein polymerization, surface drying of meat during storage, and oxidative damage to proteins [68,69]. Similar results were reported by Kaewprachu et al. (2017), who used catechin-Kradon extract in a protein-based myofibrillar film to extend the shelf life of Thunnus thynnus fish slices and observed an increase in hardness [68].

### 3.11 Smart label

**Fig. 11:**
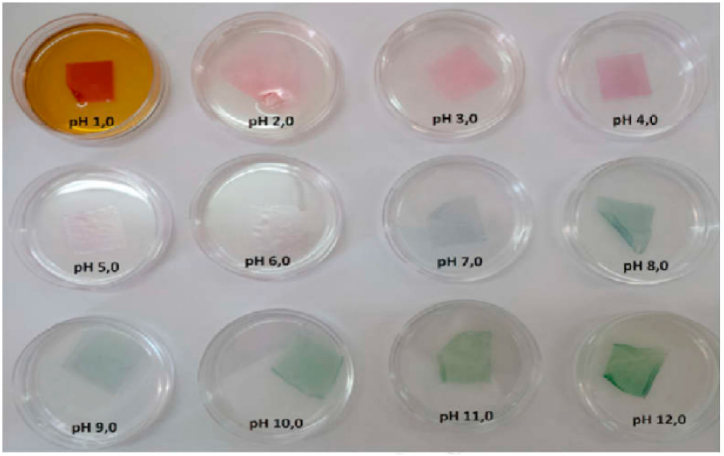
Anthocyanin indicator color change under acidic and basic conditions.

Fig. 11 displays the pH sensor color range from red to green in the pH range of 1–12. The red color indicates pH = 1, and the pink color indicates pH = 1.2, while the purple color indicates pH = 4, the white color indicates pH = 5.6, the gray color indicates pH = 7, and the green color indicates pH = 9–12. The results obtained in this study demonstrate that the changes in pH sensor color are consistent with the anthocyanin structure. Under acidic conditions, anthocyanins convert to a cationic flavylium, followed by a purple anthocyanidin quinoid at pH less than 7 and finally to a blue-dark ionized anthocyanin under alkaline conditions. Similar findings have been reported for pH sensors in cellulose-based smart food packaging containing anthocyanins from red cabbage [70,71]. Otálora González et al. (2021), stated that by adding betalin as an anthocyanin-containing compound to the edible film based on cassava starch, it leads to a change in the color of the smart tag due to the spoilage of fish muscles [72]. Firmansyah et al. (2021), also reported that pyranoflavin-cellulose acetate films containing purple cabbage anthocyanin can be a product that helps people to see the freshness of beef very easily; In this research, smart tags were made with a mixture of chitosan, polyvinyl alcohol and CMC. Meat freshness level parameters are based on pH and total microbial testing on beef; used these parameters to investigate changes in meat quality in packaging. Fresh beef has a pH between 6.7 and 7.2 and then the pH drops to 5.4–5.5 (the final pH of the beef) and then the beef tends to increase the pH (the spoilage process). In this pH range, the color change of the smart label indicator was divided into three categories, and then the state of beef (fresh, fresh and not fresh) was determined based on this [73].

## 4 Conclusion

This study aimed to investigate the potential of red beet anthocyanin extract as a pH indicator in smart packaging based on sodium alginate/chitosan, to evaluate its effect on the microbiological load of chicken fillet. Results from both chemical and microbiological tests revealed that the incorporation of red beet anthocyanin extract into the smart film led to a significant increase in pH, peroxide, nitrogenous compounds, and thiobarbituric acid index of the tested chicken fillet sample during storage time. However, this increase was comparatively less than that observed in the control and samples without anthocyanin extract (P < 0.05). Moreover, the microbiological load, including the total microbial count and coagulase-positive Staphylococcus aureus, increased during the storage time, but the rate of increase was slower in the sample packed with film containing red beet anthocyanin extract. Furthermore, the hardness of the fillet in the film containing red beet anthocyanin extract exhibited a greater increase over time compared to other treatments (P < 0.05). Overall, the incorporation of red beet anthocyanin extract in the packaging film resulted in the improvement of the chemical, microbiological, and textural properties of the tested chicken fillet. Also, studies and reviews show that the addition of anthocyanin compounds as a powerful, easy tool and has countless potential advantages such as naturalness, ease of access, wide distribution in nature, high sensitivity, cheapness, non-toxicity, and high coloring ability on the pH change, they are a good alternative to artificial colors in the development of smart packaging to monitor the freshness of food products; But despite their numerous advantages, their use in industry is hindered due to their low stability under hard conditions such as light, temperature, oxygen and enzymes.

## Author contribution statement

**Milad Ranjbar**: Performed the experiments; Wrote the paper; **Mohammad Hossein Azizi Tabrizzad**: Conceived and designed the experiments; Wrote the paper; **Gholamhassan Asadi**: Analyzed and interpreted the data; Contributed reagents, materials, analysis tools or data; **Hamed Ahari**: Conceived and designed the experiments; Contributed reagents, materials, analysis tools or data.

## Data availability statement

Data will be made available on request.

## Declaration of competing interest

The authors declare that they have no known competing financial interests or personal relationships that could have appeared to influence the work reported in this paper.
